# The association between reproductive history and the multidimensional health of older adults in rural China and its gender differences: Evidence from the Chinese longitudinal healthy longevity survey

**DOI:** 10.3389/fpubh.2022.952671

**Published:** 2022-07-27

**Authors:** Changyong Yu, Hang Liang, Boyu Wang, Fei Liang, Erpeng Liu, Nan Xiang

**Affiliations:** ^1^School of Public Administration, Zhongnan University of Economics and Law, Wuhan, China; ^2^Policy Research Center, Ministry of Civil Affairs of China, Beijing, China; ^3^Institute of Income Distribution and Public Finance, School of Public Finance and Taxation, Zhongnan University of Economics and Law, Wuhan, China

**Keywords:** reproductive history, the multidimensional health, gender differences, older adults, cognitive function, activities of daily living, symptoms of depression

## Abstract

**Background:**

Few studies have examined the association between reproductive history and the multidimensional health of older adults with more diverse reproductive histories and poorer health status in rural China. The purpose of this study is to explore the effect of parity, sex ratio of children and late childbearing on multidimensional health and its gender differences.

**Methods:**

The analytical sample consisted of 3,377 older adults in rural China who participated in the Chinese Longitudinal Healthy Longevity Survey (CLHLS) in 2018. Linear regression models were applied to estimate the relationship between reproductive history and multidimensional health, with separate models for each indicator of health outcomes.

**Results:**

Older adults in rural areas with greater parity were more likely to have better cognitive function (β = 0.409, 95% CI: 0.255–0.563), fewer Activities of Daily Living (ADL) limitations (β = −0.085, 95% CI: −0.137 to −0.034) and symptoms of depression (β = −0.396, 95% CI: −0.577 to −0.216). The social mechanism of intergenerational support from children later in life partly explained the positive effect of parity. Late childbearing had negative effects on cognitive function (β = −1.220, 95% CI: −1.895 to −0.545), ADL (β = 0.253, 95% CI: 0.028–0.478) and symptoms of depression (β = 1.025, 95% CI: 0.237–1.812). Women were more likely to be influenced by the positive effect of parity; the association between late childbearing and health was only significant in the male group.

**Conclusions:**

Parity and late childbearing are associated with cognitive function, activities of daily living, and symptoms of depression in the older adults in rural China. Older adults with more children might be in better health, and this finding is especially significant in women. However, late childbearing had a negative effect on multidimensional health, especially for men. The social mechanism and gender differences between reproductive history and health need to be further explored.

## Introduction

The world population is rapidly aging, and China is facing the largest and fastest growth in population aging. In 2020, there were 260 million older adults over the age of 60 years in China, accounting for 18.7% of the total population (1). It is estimated that by 2050, the number of older adults over the age of 60 years will increase to a peak of 488 million in China, representing 35.6% of the total population (2). In the process of population aging in China, rural population has an increasingly higher proportion of older adults than the urban (3). According to the Fifth and Sixth National Population Censuses, the population of older adults in rural areas is 15.57 and 44.21 million greater than the population of older adults in urban regions, respectively (4, 5). Improving health status of the older adults in rural areas is an important task to deal with the aging population.

China is an unhealthy aging society. According to the Fourth Sampling Survey on the Living Conditions of Urban and Rural Older People in China in 2015, 31.16% of older adults over the age of 60 years suffered from more than one chronic disease, and 30.27% of the older adults in rural areas reported poor self-rated health. The proportion of partially and completely disabled older adults was 16.5%, and the proportion of older adults who are psychologically lonely made up 30.3% in rural areas (6). The Fourth National Health Service Survey in China in 2003 (7) indicated the prevalence of disability in the population aged 60 years and above was 10.6%, which increased to 16.9% in 2008. The prevalence rates of Activities of Daily Living (ADL) disability appear to be much lower in some developed countries: 5% in Canada, 7% in France, 9% in Italy, and 7% in Sweden when age-standardized (8). Due to China's large population, the total number of disabled older adults will far exceed that of other countries. These data provide strong evidence for the judgment that China is an unhealthy aging society. Given the prevalence rate of ADL disability is significantly higher in rural areas (9), it can be seen that the health status of older adults in rural China is in bad condition. Exploring the determinants of health among the older adults in rural areas and formulating appropriate intervention policies should be the first task for China to address the challenges of the aging population.

In fact, China is a society with a profound tradition of “family culture,” reproduction is a life-course event that most Chinese people experience (10). Traditionally, infertility is considered to be one of the most unfilial behavior. Having no children means that nobody inherits property, status, family name, which are of great importance for the Chinese people. Thus, the Chinese people hold a strong belief in the concept of “the more sons, the more blessings” (11). According to Chinese Longitudinal Healthy Longevity Survey in 2018, 97.5% of the participants had at least one child. Today's older adults, most of who were born around the 1950 s, have an average of 3.0–3.5 children (6). The one-child policy, which started in the 1970 s to reduce and control births, has been implemented more loosely in rural than urban areas. As a result, older adults in rural areas have, on an average, 0.5 more children and 0.3 more boys than their urban counterparts. The relationship between reproductive history and health in older adults is an important issue in the study of life course theory. The health outcome of the older adults is a long-term accumulation process, and the early reproductive history has an important influence on the health status of the older adults in later life. To explore the relationship between reproductive history and health is helpful to guide policy direction of reproductive health service and healthy aging.

Many longitudinal studies on the direct effect of reproduction on health have shown that every life course and event from pregnancy onwards has a significant impact on health and wellbeing later in life (10, 12). The “maternal depletion theory” believes that since women are the biological undertaker of reproduction, the processes of pregnancy, childbirth, abortion and breastfeeding have more direct and greater impacts on women's current and long-term physical and mental health, especially women in developing countries with lower socioeconomic status (13–15). Some studies have shown that having more children increases the risk of chronic diseases and disability in elderly women (16–18), whereas others have suggested that women with more children have a lower risk of breast cancer and ovarian cancer (19). In countries where parents receive generous support from the state, greater parity is associated with better health for both men and women (20). A study based on US National Longitudinal Survey of Youth in 1979 showed that late childbearing is associated with better language and cognitive function (21). However, a few studies have shown that late childbirth may cause physical health problems, and older age at last childbirth increases the risk of women suffering from reproductive diseases such as breast cancer (22, 23). Previous research indicated that the mean age at childbearing of China experienced a decrease between 1990 and 1995, followed by a sustaining growth afterwards (24). 17.28% of women gave birth at an advanced maternal age (≥35) in rural areas, while 12.62% of women in urban areas in 2019 (25). Thus, older adults in rural China had a more complicated reproductive history. The relationship between reproductive behavior and the health of older adults is very complex and needs further analysis (26).

With in-depth exploration of the social mechanism of the indirect effect of reproductive history on health, the focus has been extended to both genders and not confined to elderly women (27, 28). Although the biological undertaker of birth are women, both men and women invest a large amount of economic and emotional resources in bringing up their children, and childbirth will change individual's behavior, psychology, social norms, and economic responsibilities for both men and women (10, 29, 30). Especially in counties where men have the economic responsibilities, they have more financial responsibility and stress and are more likely to smoke, drink, and develop other unhealthy lifestyles (31–33). Of course, it should also be noted that from the perspective of social mechanisms, reproduction does not necessarily have a negative effect on the health of older adults because children also provide financial, daily care, spiritual comfort, and other support to older adults, which helps improve health status later in life (34, 35).

A few studies have explored the effects of reproductive history on the health of older adults in China from both direct and indirect perspectives. In terms of direct effects, elderly women with many children are more likely to suffer from impairment in ADL, poorer self-rated health, and higher mortality (36, 37). Studies indicate that when the number of children ever born or living children is >5, the lifespan of elderly women tends to be longer (38). From the perspective of indirect effects, family provides the most important support to older adults in China, especially in rural areas. Adult children tend to be the main source of external support, such as economic needs, daily care, and spiritual comfort for older adults. Thus, reproduction has a positive, indirect impact on the health of older adults (39).

In general, available research on the effect of reproductive history on the health of older adults in rural China is still limited. Most of the studies concentrated on women and social mechanisms by which reproduction affects the health of the older adults from an indirect perspective (such as economic, caring, and spiritual comfort from children). Few studies have explored the direct impact of reproductive history, and the relevant conclusions are not consistent. Life course theory suggests that early behavior or life events could have a direct or indirect impact on the health status of older adults (40). For the older adults with poor health in rural China, reproduction is a typical and almost inevitable life course event, and its impact on the health of the older adults in rural China is an important topic worth exploring.

The influence of reproductive history on the multidimensional health of older adults includes biological mechanism and social mechanism. Biological mechanism can be regarded as a direct effect of fertility, which refers to the direct physiological impact on women as the bearer of fertility behavior. Specifically, having more children increases the risk of chronic diseases and disability in elderly women (16–18); late childbirth may cause physical health problems and older age at last childbirth increases the risk of women suffering from reproductive diseases such as breast cancer (22, 23). Elderly women with many children are more likely to suffer from impairment in ADL, poorer self-rated health, and higher mortality (36, 37). However, a few studies also showed there was a positive association between reproductive history and multidimensional health. More children is associated with lower risk of breast cancer and ovarian cancer, and better health for both men and women; the lifespan of elderly women that have children more than five tends to be longer (19, 20, 38). Social mechanism refers to the indirect effects of reproductive history on multidimensional health. For example, raising children requires more time and energy, which affects their physical and mental health, and adult children's intergenerational support too may improve their health. Some studies indicated that men have more financial responsibility and stress of bringing up their children and are more likely to smoke, drink, and develop other unhealthy lifestyles (31–33). However, children also provide financial support, daily care, spiritual comfort, and other support to older adults, which help improve health status later in life (34, 35).

Compared with previous studies, there are several contributions of this paper. First, both the direct and indirect effects of reproductive history on the health of the older adults in rural China are investigated in our research. Second, the study population is the older adults of rural China with a more diverse reproductive history and poorer health status, and both women and men are included in our study. Third, based on the parity and timing of reproduction, adding the perspective of sex ratio of born children, we comprehensively investigate the effects of reproductive history on multidimensional health (including cognitive function, physical health, and mental health) of the older adults in rural areas of China.

## Methods

### Data source and study sample

The data were derived from the Chinese Longitudinal Healthy Longevity Survey (CLHLS), a nationally representative survey jointly performed by the Center for Healthy Aging and Development Studies at Peking University and Duke University. The CLHLS was first conducted in 1998, followed by seven waves of surveys in 2000, 2002, 2005, 2008, 2011, 2014, and 2018 from 22 sample areas in 31 provincial administrative units. The population of the surveyed region accounts for 85.3% of the total population of the country, which can be regarded as a nationally representative sample (41). Additional details, such as the sampling design, sampling weight, and assessment of data quality, can be found in previous studies (42, 43). We used the latest cross-sectional data from the CLHLS in 2018. Only those individuals aged 60 years and above in rural areas with at least one child were selected for the present analysis. After filtering for missing values, outliers, etc., the present study sample included 3,377 older adults in rural areas.

## Measurements

### Multidimensional health

Three indicators of health outcomes, cognitive function, ADL, and depressive symptoms were used as dependent variables in our research.

Cognitive function was assessed by 24 items modified from the Mini-Mental State Examination scale (MMSE) (44), which has been proven to be reliable and effective with Chinese older adults (43, 45). It contains a brief set of components to assess orientation, concentration, memory, and language, with total scores ranging from 0 to 30. Higher scores indicate better cognitive function.

The Katz scale was adopted to evaluate ADL, which included six items: bathing, dressing, bathroom use, movement indoors, continence, and eating. Respondents were asked to rate their ability to engage in these ADLs using a three-point scale: (1) not difficult at all, (2) slightly difficult, and (3) unable to do the task. The total scores ranged from 3 to 18, and higher scores indicated more limited functioning for the respondent.

Depressive symptoms were assessed by the Center for Epidemiologic Studies Depression Scale (CES-D) (46). The scale consists of 9 items, including 7 negative feeling items (e.g., lonely and isolated) and 2 positive feeling items (e.g., hopeful about the future). Respondents reported how often they had a particular feeling (1 = always, 2 = often, 3 = sometimes, 4 = seldom, 5 = never). The total scores ranged from 5 to 45, and higher scores indicated more depressive symptoms after reversing the coding of the 7 negative items.

### Reproductive history

Information about reproductive history was collected in CLHLS through the following measures. Parity was measured by the number of biological children reported by the respondents, including deceased children. The participants further reported the number of sons that were ever born so we could calculate the sex ratio by dividing the number of sons by parity. Women who give birth over the age of 40 are considered advanced maternal age (26). We define late childbearing as a binary variable, which is set to 0 if the last birth was before the age of 40 or 1 if the last birth was at 40 or above.

### Intergenerational support

To capture the indirect effects of reproduction, we were concerned about intergenerational support from children, which might be beneficial to older adults, and included three aspects: economic support, daily care, and spiritual comfort. Economic support is measured by the total value of financial transfers and in-kind assets transfers those older adults received last year from their children. Daily care was assessed by the hours that children spend caring for older adults in the last week. Spiritual comfort was the total score of the following three questions: did you talk to your children frequently? (1 = yes; 0 = no); did you first talk to your children when you needed to tell someone about your thoughts? (1 = yes; 0 = no); did you first ask your children for help when you had problems/difficulties? (1 = yes; 0 = no).

### Covariates

All analyses presented here included a series of covariates associated with health as follows according to previous studies (47–51), which provide a good basis for the selection of control variables in this paper: (1) demographic characteristics, including gender, age, education, and marital status; (2) family factors, including family income, number of persons living with you, and housing condition; (3) social support, including medical insurance and the total amount of available social services the participant reported; and (4) lifestyle, including whether they had smoked/drank/exercised in the past (1 = yes; 0 = no) were also queried. Family income was measured by the logarithm of income per capita in the household last year. Housing condition was a dummy variable of whether your home frequently has a mildew odor or a musty smell.

### Statistical analysis

We first present the characteristics of the Chi-square tests for categorical variables and *T*-tests for continuous variables were used to test the significance of gender comparisons. Then, the multidimensional health of participants in groups with different reproductive histories was reported. *T*-tests and analyses of variance (ANOVAs) were performed to compare the health outcomes between different reproductive history groups. Finally, linear regression models were used to estimate the relationship between reproductive history and multidimensional health, with separate models for each indicator of health outcomes. Reproductive history and intergenerational support were separately included in the two models to distinguish between direct and indirect effects. Then, both reproductive history and intergenerational support were included in the third model. Furthermore, we present the gender-stratified results to explore gender differences in the relationship between reproductive history and multidimensional health. Data management and statistical analyses were performed using Stata 15 statistical software (Stata Corp, College Station, TX, USA).

## Results

Descriptive characteristics of the study participants (*N* = 3,377) by gender are shown in Table 1. There were 2,039 (60.38%) women and 1,338 (39.62%) men who participated in the study. The average age of the respondents was approximately 85 years. The women in the study population were older than the men by 4 years (*p* < 0.001), had fewer years of education (*p* < 0.001), lower family income (*p* = 0.054), were more likely to be widowed (*p* < 0.001), and were more likely to be without medical insurance (*p* = 0.002). Men tended to smoke (*p* < 0.001), drink (*p* < 0.001), and exercise (*p* < 0.001) in the past. For multidimensional health, women were more likely to be in worse health in terms of cognitive function (*p* < 0.001), ADL (*p* < 0.001), and depressive symptoms (*p* < 0.001).

**Table 1:** Descriptive statistics.

**Variables**	**Measurement**	***N*** **(%)/Mean (SD)**			***P*** **-value**
		**Total** **(*N* = 3,377)**	**Women** **(*N* = 2,039)**	**Men** **(*N* = 1,338)**	
Age	Continuous measurement	85.42 (11.67)	87.16 (11.93)	82.76 (10.75)	<0.001
Education (years)	Continuous measurement	2.14 (3.20)	1.02 (2.22)	3.86 (3.66)	<0.001
Marital status	Married = 1	1,283 (37.99)	532 (26.09)	751 (56.13)	<0.001
	Other = 0	2,094 (62.01)	1,507 (73.91)	587 (43.87)	
Family income (ln)	Continuous measurement	9.56 (1.77)	9.517 (1.82)	9.64 (1.68)	0.054
Number of persons living with you	Continuous measurement	2.18 (2.03)	2.20 (2.00)	2.15 (2.07)	0.492
Housing condition	Have musty smell = 1	553 (16.38)	341 (16.72)	212 (15.84)	0.499
	No musty smell = 0	2,824 (83.62)	1,698 (83.28)	1,126 (84.16)	
Medical insurance	Yes = 1	2,701 (79.98)	1,666 (81.71)	1,035 (77.35)	0.002
	No = 0	676 (20.02)	373 (18.29)	303 (22.65)	
Social service	Continuous measurement	1.22 (1.45)	1.21 (1.46)	1.24 (1.45)	0.512
Smoked	Yes = 1	964 (28.55)	156 (7.65)	808 (60.39)	<0.001
	No = 0	2,413 (71.45)	1,883 (92.34)	530 (39.61)	
Drank	Yes = 1	822 (24.34)	208 (10.20)	614 (45.89)	<0.001
	No = 0	2,555 (75.66)	1,831 (89.80)	724 (54.11)	
Exercised	Yes = 1	757 (22.42)	391 (19.18)	366 (27.35)	<0.001
	No = 0	2,620 (77.58)	1,648 (80.82)	972 (72.65)	
Cognitive function	Continuous measurement	21.68 (9.60)	19.89 (10.06)	24.40 (8.14)	<0.001
Activities of daily living (ADL)	Continuous measurement	7.41 (2.80)	7.75 (3.08)	6.88 (2.21)	<0.001
Depressive symptoms	Continuous measurement	23.66 (9.79)	24.99 (10.34)	21.63 (8.50)	<0.001

“*N (%)*” means the sample size of a particular category in a categorical variable and its proportion in the total sample. “Mean (SD)” represents the sample mean of a continuous variable and its standard error.

Table 2 displays the distributions by reproductive history variables and multidimensional health of participants in groups with different reproductive histories. The participants who had 3 to 6 children (categories: 3–4 and 5–6 children) accounted for a large proportion (70.75%) of the whole sample. There were significant differences in cognitive function (*p* < 0.001), ADLs (*p* < 0.001), and depressive symptoms (*p* < 0.001) of older adults with different numbers of children. In brief, the participants with greater parity were more likely to have worse health outcomes. In terms of sex ratio, 43.94% of the sample had more boys, and 34.82% of the sample had more girls. No significant differences were found in the relationship between health outcomes and the sex ratio of their children except cognitive function (*p* = 0.075). Nearly a quarter of older adults had their last birth at 40 years or above, and they had worse conditions of cognitive function (*p* < 0.001), ADL (*p* < 0.001), and depressive symptoms (*p* < 0.001).

**Table 2:** Multidimensional health of older adults with different reproductive history (*N* = 3,377).

	**Number (%)**	**Cognitive function**		**ADL**		**Depressive symptoms**	
		**Mean (SD)**	***p*** **-value**	**Mean (SD)**	***p*** **-value**	**Mean (SD)**	***p*** **-value**
Parity							
1–2	565 (16.73)	23.384 (9.314)	<0.001	7.065 (2.492)	<0.001	22.149 (9.783)	<0.001
3–4	1,372 (40.63)	23.007 (9.013)		7.128 (2.560)		23.023 (9.290)	
5–6	1,017 (30.12)	20.372 (9.781)		7.679 (3.004)		24.572 (9.970)	
7+	423 (12.53)	18.217 (10.078)		8.116 (3.226)		25.551 (10.429)	
Sex ratio of children							
More sons	1,484 (43.94)	21.341 (9.556)	0.075	7.426 (2.822)	0.111	23.854 (9.861)	0.402
Equal number	717 (21.23)	22.332 (9.484)		7.222 (2.627)		23.252 (9.764)	
More daughters	1,176 (34.82)	21.701 (9.716)		7.497 (2.876)		23.663 (9.710)	
Last birth
After 40 years	792 (23.45)	17.775 (10.460)	<0.001	8.226 (3.330)	<0.001	26.380 (10.844)	<0.001
Before 40 years	2,585 (76.55)	22.872 (8.991)		7.156 (2.568)		22.826 (9.284)	

Table 3 shows the results from linear regressions assessing the relationship between reproductive history and multidimensional health after adjusting for covariables. The three health-related outcomes were significantly associated with parity and late childbearing. Older adults in rural areas with greater parity were more likely to have better cognitive function (β = 0.409, 95% CI: 0.255–0.563), fewer ADL limitations (β = −0.085, 95% CI: −0.137 to −0.034), and fewer depressive symptoms (β = −0.396, 95% CI: −0.577 to −0.216). Late childbearing also had a negative effect on cognitive function (β = −1.220, 95% CI: −1.895 to −0.545), ADL (β = 0.253, 95% CI: 0.028–0.478), and depression symptoms (β = 1.025, 95% CI: 0.237–1.812). To capture the indirect effect, we further explored how intergenerational support from children might affect the health of older adults in rural areas in Model 2. The results showed that economic support had a positive effect on health in terms of cognitive function (β = 0.112, 95% CI: 0.041–0.184) and depressive symptoms (β = −0.110, 95% CI: −0.196 to −0.025). Spiritual comfort from children might benefit their parents' health. However, daily care was negatively associated with cognitive function (β = −0.050, 95% CI: −0.055 to −0.044), ADL (β = 0.0263, 95% CI: 0.025–0.028), and depressive symptoms (β = 0.041, 95% CI: 0.035–0.048). The Model 3 included all variables. When intergenerational support was considered, the relationship between parity and health outcomes still passed the significance test with a slight reduction, and the estimated coefficient decreased compared with the Model 1. Late childbearing negatively affected cognitive function (β = −1.071, 95% CI: −1.715 to −0.428) and depressive symptoms (β = 0.927, 95% CI: 0.162–1.692), but the association between late childbearing and ADL became non-significant.

**Table 3:** Linear regression models examining the association between reproductive history and multidimensional health (*N* = 3,377).

	**Model 1 direct effect**	**Model 2 indirect effect**	**Model 3 overall effect**
	***β*** **(95% CI)**	***β*** **(95% CI)**	***β*** **(95% CI)**
**Dependent variable: cognitive function**			
Parity	0.409^***^ (0.255–0.563)		0.312^***^ (0.163–0.460)
Sex ratio	0.388 (−0.623 to 1.401)		0.211 (−0.755 to 1.177)
Late childbearing	−1.220^***^ (−1.895 to −0.545)		−1.071^**^ (−1.715 to −0.428)
Economic support		0.112^**^ (0.041–0.184)	0.097^***^ (0.0244–0.169)
Daily care		−0.050^***^ (−0.055 to −0.044)	−0.049^**^ (−0.055 to −0.044)
Spiritual comfort		0.940^***^ (0.674–1.206)	0.924^***^ (0.658–1.190)
Constant	54.330^***^ (51.183–57.476)	49.784^***^ (46.800–52.770)	48.721^***^ (45.632–51.810)
**Dependent variable: ADL**			
Parity	−0.085^**^ (−0.137 to −0.034)		−0.054^*^ (−0.101 to −0.008)
Sex ratio	−0.308 (−0.646 to 0.029)		−0.192 (−0.494 to 0.109)
Late childbearing	0.253^*^ (0.028–0.478)		0.162 (−0.038 to 0.363)
Economic support		−0.007 (−0.030 to 0.015)	−0.005 (−0.028 to 0.017)
Daily care		0.026^***^ (0.025–0.028)	0.026^***^ (0.024–0.028)
Spiritual comfort		−0.198^***^ (−0.281 to −0.115)	−0.194^***^ (−0.277 to −0.111)
Constant	−0.056^***^ (−1.106 to −0.993)	1.934^***^ (1.004–2.864)	2.203^***^ (1.238–3.167)
**Dependent variable: Depressive symptoms**			
Parity	−0.396^***^ (−0.577 to −0.216)		−0.296^**^ (−0.472 to −0.119)
Sex ratio	−0.815 (−1.995 to 0.365)		−0.656 (−1.805 to 0.493)
Late childbearing	1.025^*^ (0.237–1.812)		0.927^*^ (0.162–1.692)
Economic support		−0.110^*^ (−0.196 to −0.025)	−0.098^*^ (−0.184 to −0.012)
Daily care		0.041^***^ (0.035–0.048)	0.041^***^ (0.0339–0.047)
Spiritual comfort		−1.248^***^ (−1.564 to −0.932)	−1.229^***^ (−1.545 to −0.913)
Constant	6.625^***^ (2.956–10.290)	10.730^***^ (7.184–14.280)	11.970^***^ (8.295–15.64)

The 95% confidence intervals are in parentheses.

* p < 0.05;

** p < 0.01;

*** p < 0.001.

Table 4 displays the results of the gender stratification after controlling for the indirect effect. Women with greater parity were more likely to have better cognitive function (β = 0.339, 95% CI: 0.149–0.528) and fewer depressive symptoms (β = −0.297, 95% CI: −0.525 to −0.069), but these relationships did not pass the significance test in men. Men whose last child was born when they were 40 years old or older experienced cognitive decline (β = −1.926, 95% CI: −2.872 to −0.980) and had more ADL limitations (β = 0.455, 95% CI: 0.177–0.733).

**Table 4:** Linear regression models examining the association between reproductive history and multidimensional health by gender.

	**Women (*N* = 2,039)**		**Men (*N* = 1,338)**	
	***β***	**95% CI**	***β***	**95% CI**
**Dependent variable: Cognitive function**				
Parity	0.339^***^	(0.149–0.528)	0.151	(−0.090 to 0.391)
Sex ratio	−0.335	(−1.660 to 0.991)	0.564	(−0.815 to 1.943)
Late childbearing	−0.537	(−1.400 to 0.327)	−1.926^***^	(−2.872 to −0.980)
Constant	53.015^***^	(48.861–57.169)	42.973^***^	(38.223–47.723)
**Dependent variable: ADL**				
Parity	−0.060	(−0.121 to 0.001)	−0.016	(−0.087 to 0.054)
Sex ratio	−0.180	(−0.605 to 0.245)	−0.025	(−0.430 to 0.380)
Late childbearing	0.005	(−0.272 to 0.282)	0.455^**^	(0.177–0.733)
Constant	1.377^**^	(0.045–2.709)	3.348^***^	(1.954–4.741)
**Dependent variable: Depressive symptoms**				
Parity	−0.297^*^	(−0.525 to −0.069)	−0.168	(−0.449 to 0.113)
Sex ratio	−0.070	(−1.663 to 1.523)	−0.998	(−2.611 to 0.614)
Late childbearing	0.587	(−0.451 to 1.625)	1.406^*^	(0.299–2.513)
Constant	9.137^***^	(4.146–14.129)	15.170^***^	(9.611–20.722)

The 95% confidence intervals are in parentheses.

* p < 0.05;

** p < 0.01;

*** p < 0.001.

## Discussion

In this study, we investigated associations between reproductive history and multidimensional health (cognitive function, ADL, and depressive symptoms) using nationally representative cross-sectional data from older adults in rural China. The results indicated that greater parity was associated with better cognitive function, fewer ADL limitations, and fewer depressive symptoms. This finding was not consistent with several studies (16–18, 27), but the positive effect of greater parity on health was also found in a longitudinal study that showed that elderly women who had five or more children were more likely to live longer in China (38). Having more children means that pregnancy and breastfeeding continue uninterrupted, and it prolongs the cycle of female estrogen in production and stimulates the female biological system, thus promoting survival and health among women (52). In addition, the relationship between parity and health suggests that higher than average reproductive potential may be related to better health (14). Moreover, parity is a positive factor affecting happiness and social support (53, 54), and older adults with more children might be healthier.

To further explore the influence mechanisms, we considered intergenerational support (economic support, daily care, and spiritual comfort from children) to capture the indirect effect of reproduction. The coefficient of parity on multidimensional health is reduced but still significant. This result suggested that the positive effect of parity could be partly explained by support from children. Previous studies also confirmed that older adults with more intergenerational support were likely to have fewer depressive symptoms and higher life satisfaction (35, 36, 55). The salience of different mechanisms linked to health may depend on the reproductive culture in different countries (26). China is a country that advocates for filial piety. It is children's responsibility to take care of their parents later in life. Thus, raising children means a long-term investment in the health of older adults in China (56). The more children they have, the more resources they can obtain in their old age. Previous study found a positive “supporting effect” of the parity on parental health. That is, the availability of additional children in old age has a beneficial effect on health during that time (57). In addition, the regression model showed that economic support and spiritual comfort benefit older adults, whereas only daily care had a negative effect on multidimensional health. This finding might be explained by reverse causality, since older adults with cognitive impairment or ADL limitations might need more daily care.

We found no evidence of an association between the sex ratio of children and the multidimensional health among older adults in rural areas, but late childbearing was associated with worse cognitive function and more depressive symptoms. Similar results have been found in several studies that having a child after age 35 years was detrimental to later-life health (22, 58). In terms of mental health, late childbearing may be associated with greater depression (21).

We finally explored gender differences in the relationship between reproductive history and multidimensional health. Women who gave birth to more children were more likely to have better cognitive function and fewer depressive symptoms. The association between late childbearing and health was only significant in males. It is said that the impact of reproduction on men's health mainly occurs through external social factors, whereas women are influenced by both internal (biological) and external (social) mechanisms (59, 60). In China, men are primarily responsible financially for childbearing. Late childbirth might result in a heavy burden on men, and they would spend more time working and earning money. The working characteristics of men in rural areas, whether they stay in rural areas to engage in agricultural labor or go out to work in urban regions, such as construction workers, are mainly physical labor. The burden of late childbirth has a greater impact on men's health. Therefore, being overworked for a long time may lead to worse health.

Another point should be explained from Table 2. The health of older adults with more children was worse than their counterparts, whereas greater parity resulted in better health after controlling for all covariates in Table 4. This contradictory result might be caused by the fact that older adults who give birth to more children tend to be older. In our sample, only 9.52% of individuals aged 60–70 years had more than five children, but the proportion of participants over 90 years who had five children was 52.25%. It is universally acknowledged that older adults tend to be in worse health. Consequently, when other variables were not controlled, the parity was negatively correlated with health.

The results of regression analysis also showed that age had significant negative impact on the cognitive function (*p* < 0.001), ADL (*p* < 0.001), and symptoms of depression (*p* < 0.001), which suggest that older adults tend to have worse cognitive function, ADL, and symptoms of depression. This finding was consistent with several studies (61–63). As we all know, age plays a fundamental role in the health of older adults. With the increase of age, the function of the body organs of older adults gradually declines, which is reflected by the worse cognitive function, more ADL limitations. and worse symptoms of depression. Therefore, attention should be paid to the health of the oldest-old and provide targeted measures to improve their health.

Nevertheless, the results should be interpreted cautiously due to several limitations. We used only cross-sectional data but not longitudinal data. There may be covariates that affect reproductive decisions and health that were not included in the present study, such as the health status when they were pregnant. Women who give birth to more children might be healthier than others when they are young. In addition, we fail to engage with the possibility of survival bias. Chinese Longitudinal Healthy Longevity Survey (CLHLS) began in 2002. During the following years in 2005, 2008, 2011, 2014, and 2018, there were some lost to follow-ups, deaths, and newly added samples. Since the data of 2018 CLHLS used in our study only contains older adults that survived to 2018, we do not have information for adults that died before this year. It is possible that there is an unobserved mechanism that affects both fertility and health and survival. Finally, for the lack of information in the questionnaire, the paper failed to include some control variables into regression analysis, such as current location of the children. If possible, we will discuss it further in the future.

## Conclusions

This study analyzed the association between reproductive history and the multidimensional health of older adults in rural China. Based on cross-sectional data from the CLHLS in 2018, we found that greater parity was associated with better cognitive function, fewer ADL limitations, and fewer depressive symptoms in older adults in rural China. Intergenerational support, including economic support, daily care, and spiritual comfort, partly explained the positive effect of parity. Late childbearing was associated with worse cognitive function and more depressive symptoms. There were gender differences in these relationships. Women were more likely to be influenced by the positive effect of parity, whereas the association between late childbearing and health was only significant in males.

## Data availability statement

Publicly available datasets were analyzed in this study. This data can be found here: https://opendata.pku.edu.cn/dataverse/CHADS.

## Ethics statement

Written informed consent was obtained from the individual(s) for the publication of any potentially identifiable images or data included in this article.

## Author contributions

CY: conceptualization, formal analysis, and funding acquisition. HL: software, writing—review, and editing. FL: writing—review and editing. BW: methodology. EL: funding acquisition, writing—review, and editing. NX: formal analysis, methodology, and writing—original draft. All authors contributed to the article and approved the submitted version.

## Funding

This study was supported by the Higher Education Discipline Innovation Project (Plan 111) of Ministry of Education of China, and the fund name is Income Distribution and Public Finance (grant number: B20084) to EL. The funder website is http://iidpf.zuel.edu.cn/column/yjyjj. This study was also supported by the National Office for Philosophy and Social Sciences, and the fund name is Study on the Effect and Mechanism of Chronic Diseases on Rural Aged Poverty (grant number: 18BSH050) to CY. The funder website is http://www.nopss.gov.cn/. This study was also supported by the University-level scientific research project of Zhongnan University of Economics and Law, and the fund name is Study on the Governance Mechanism of Rural Elderly Relative Poverty from the Perspective of Chronic Diseases (grant number: 31522241202) to CY. The funders had no involvement in study design, data collection, statistical analysis, and manuscript writing.

## Conflict of interest

The authors declare that the research was conducted in the absence of any commercial or financial relationships that could be construed as a potential conflict of interest.

## Publisher's note

All claims expressed in this article are solely those of the authors and do not necessarily represent those of their affiliated organizations, or those of the publisher, the editors and the reviewers. Any product that may be evaluated in this article, or claim that may be made by its manufacturer, is not guaranteed or endorsed by the publisher.
